# *Serendipita indica* promotes P acquisition and growth in tea seedlings under P deficit conditions by increasing cytokinins and indoleacetic acid and phosphate transporter gene expression

**DOI:** 10.3389/fpls.2023.1146182

**Published:** 2023-03-17

**Authors:** Zi-Yi Rong, An-Qi Lei, Qiang-Sheng Wu, Anoop Kumar Srivastava, Abeer Hashem, Elsayed Fathi Abd_Allah, Kamil Kuča, Tianyuan Yang

**Affiliations:** ^1^College of Horticulture and Gardening, Yangtze University, Jingzhou, Hubei, China; ^2^State Key Laboratory of Tea Plant Biology and Utilization, Anhui Agricultural University, Hefei, Anhui, China; ^3^Department of Chemistry, Faculty of Science, University of Hradec Kralove, Hradec Kralove, Czechia; ^4^ICAR-Central Citrus Research Institute, Nagpur, Maharashtra, India; ^5^Botany and Microbiology Department, College of Science, King Saud University, Riyadh, Saudi Arabia; ^6^Plant Production Department, College of Food and Agricultural Sciences, King Saud University, Riyadh, Saudi Arabia

**Keywords:** auxin, endophytic fungi, cash crop, nutrient deficit, phytohormone, symbiosis

## Abstract

The culturable endophytic fungus *Serendipita indica* has many beneficial effects on plants, but whether and how it affects physiological activities and phosphorus (P) acquisition of tea seedlings at low P levels is unclear. The objective of this study was to analyze the effects of inoculation with *S*. *indica* on growth, gas exchange, chlorophyll fluorescence, auxins, cytokinins, P levels, and expressions of two phosphate transporter (*PT*) genes in leaves of tea (*Camellia sinensis* L. cv. Fudingdabaicha) seedlings grown at 0.5 μM (P_0.5_) and 50 μM (P_50_) P levels. Sixteen weeks after the inoculation, *S*. *indica* colonized roots of tea seedlings, with root fungal colonization rates reaching 62.18% and 81.34% at P_0.5_ and P_50_ levels, respectively. Although plant growth behavior, leaf gas exchange, chlorophyll values, nitrogen balance index, and chlorophyll fluorescence parameters of tea seedlings were suppressed at P_0.5_ versus P_50_ levels, inoculation of *S*. *indica* mitigated the negative effects to some extent, along with more prominent promotion at P_0.5_ levels. *S*. *indica* inoculation significantly increased leaf P and indoleacetic acid concentrations at P_0.5_ and P_50_ levels and leaf isopentenyladenine, dihydrozeatin, and transzeatin concentrations at P_0.5_ levels, coupled with the reduction of indolebutyric acid at P_50_ levels. Inoculation of *S*. *indica* up-regulated the relative expression of leaf *CsPT1* at P_0.5_ and P_50_ levels and *CsPT4* at P_0.5_ levels. It is concluded that *S*. *indica* promoted P acquisition and growth in tea seedlings under P deficit conditions by increasing cytokinins and indoleacetic acid and *CsPT1* and *CsPT4* expression.

## Introduction

Phosphorus (P) is an important mineral nutrient required for plant growth, and it is involved in many metabolic processes of plants, including carbon allocation, energy transfer, photosynthesis, and respiration (Taghinasab et al., 2018). Insufficient P concentrations in plants negatively affect their metabolisms, leading to the reduction of plant productivity (Taghinasab et al., 2018). Plants can absorb inorganic phosphate from the soil around the roots through phosphate transporters (PTs) (Liao et al., 2019). The P in the soil is easily fixed, which limits the uptake of P by the roots. The uptake of soil P by the roots cannot meet the needs of plant growth (Vance et al., 2003; Wu F et al., 2019). Plants can establish a symbiotic relationship with soil arbuscular mycorrhizal fungi (AMF) in roots (Zou et al., 2021; Wang et al., 2023), in which the mycorrhizal pathway helps plants to acquire P from the soil (Deng et al., 2022; Sun RT et al., 2022). However, as obligate symbiotic fungi, AMF is unable to reproduce rapidly *in vitro* without plant roots, thereby limiting its application on a large scale in the field.

*Serendipita indica* (Sav. Verma, Aj. Varma, Rexer, G. Kost & P. Franken) M. Weiß, Waller, A. Zuccaro & Selosse (earlier *Piriformospora indica*) is an endophytic fungus isolated from the rhizosphere of desert plants in India (Cao MA et al., 2022), and *S*. *indica* can be cultured *in vitro* without plant roots (Yang et al., 2020). The fungus has important benefits in P uptake and growth of host plants (Loha et al., 2018; Yang L et al., 2021; Cao MA et al., 2022; Liu et al., 2022). In addition, *S*. *indica* has many positive effects on promoting water absorption, enhancing stress resistance, and increasing crop yield (Saddique et al., 2018; Bakhshandeh et al., 2020; Tyagi et al., 2022). Similar to AMF, *S*. *indica* also promotes plant growth performance. Wang et al. (2022) found that *S*. *indica* changed the pH value and phosphatase activities of cadmium-contaminated soil to improve the photosynthesis of soybean. Similarly, Liu BH et al. (2021) found that *S*. *indica* promoted chlorophyll content, fluorescence parameters, and antioxidant enzyme activities to improve the growth performance of walnut plants under drought stress. *S*. *indica* could establish the symbiosis with the roots of *Pleurotus orientalis*, which thus improved root architecture, net CO_2_ assimilation, and light use efficiency (Wu C et al., 2019). In addition, *S*. *indica* promoted soil acidic, neutral, alkaline, and total phosphatase activities of trifoliate orange seedlings, thus improved P acquisition (Yang L et al., 2021). Field inoculation experiments also showed that *S*. *indica* could promote the accumulation of glucose, fructose and sucrose as well as P concentrations in Newhall Navel orange fruits (Cheng et al., 2022). It means that *S*. *indica* could bring benefits to plants in many ways.

Tea (*Camellia sinensis* L.), an important cash crop in the world, is mainly planted in acidic soil and is often limited by soil P deficiency (Zheng et al., 2012; Wang et al., 2015). Studies have found that AMF improved root system architecture of tea plants and up-regulated the expression of *CsPT1* and *CsPT4* to promote the absorption of P and plant growth (Shao et al., 2021). The inability of AMF to propagate on a large scale limits its use in the field, whereas *S*. *indica* presents AMF-like properties and can be cultured *in vitro*, thus having a strong potential for large-scale application in the field (Yang L et al., 2021; Cao MA et al., 2022). At present, it is not clear whether *S*. *indica* has a positive effect on plant growth and P acquisition of tea plants, and what the underlying mechanism of the effect is.

The present study tried to analyze the effects of *S*. *indica* inoculation on the growth, leaf gas exchange, chlorophyll fluorescence parameters, nitrogen balance index, phytohormone levels, leaf P concentrations, and *PT* gene expression of tea plants grown at two P levels, in order to uncover the mechanism of *S*. *indica* promoting P acquisition of tea plants and the potential application of *S*. *indica* in field tea cultivation.

## Materials and methods

### Fungal inoculums

*S*. *indica* was provided by Professor Tian, College of Life Sciences, Yangtze University (Jingzhou, China). Fungal mass of *S*. *indica* were inoculated into solid potato glucose medium and incubated at 23 °C for 3 weeks. After the mycelium covered the medium, a small amount of sterile water was added. Subsequently, the fungus on the surface of the medium was transferred to another triangular flask using a sterile glass rod, with 500 mL sterile water to obtain a spore suspension of *S*. *indica*. The spore suspension was mixed with distilled water at a ratio of 1: 20 as the next fungal inoculums, in which the number of spores was calculated using a spectrophotometer and the concentration was 2.81 × 10^9^ CFU (colony forming units)/mL.

### Plant culture

Seeds of *C*. *sinensis* L. cv. Fudingdabaicha were provided by the Tea Research Institute, Guizhou Academy of Agricultural Science (Guiyang, China). The seeds were disinfected with 75% ethanol for 10 minutes and washed with sterile water 3 times. Then the seeds were germinated in autoclaved (121°C, 0.12 MPa, 1 h) sand in an incubator with a diurnal temperature of 28°C/20°C. After 4 weeks, the tea seedlings having two leaves were transplanted into plastic pots with an upper diameter of 18 cm, a bottom diameter of 11 cm, and a height of 15 cm. The potted substrate was an autoclaved (121°C, 0.12 MPa, 2 h) mixture of the soil and sand, with a volume ratio of 3:1. The soil characteristics were pH 5.9, Olsen-P 10.4 mg/kg, available K 38.6 mg/kg, and soil organic carbon 8.0 mg/g. Each pot contained 1.2 kg of autoclaved substrates.

*S*. *indica* inoculations were performed at the time of transplanting, where *S*. *indica*-inoculated treatment was arranged as 50 mL of above spore suspension per pot. The treatment without *S*. *indica* inoculation was applied with an equal amount of autoclaved (121°C, 0.12 MPa, 2 h) spore suspension.

Two P levels including 0.5 μM and 50 μM P were arranged according to Shao et al. (2021). P concentrations were achieved by varying the KH_2_PO_4_ levels in the Hoagland solutions, and the details had been described in Shao et al. (2021). One week after *S*. *indica* inoculation, P treatments were performed, where each pot received 80 mL of designed Hoagland solutions, once every two days, for a total of six applications.

All potted seedlings were placed in a greenhouse with a photo flux density of 948 μmol/m^2^/s, diural-night temperature of 28°C/23°C, and relative air humidity of 67%. During the experiment, each pot received 50 mL distilled water per day on non-P-treated days. The position of the experimental pots was moved weekly to avoid differences in the surrounding environment. The experiment lasted for 16 weeks from May to September 2021.

### Experimental design

This experiment consisted of two factors, including two P treatments (0.5 μM and 50 μM P) as well as two fungal inoculation treatments (*S*. *indica* inoculation and uninoculation). A total of four treatments were arranged, namely, (i) tea seedlings inoculated without *S*. *indica* under 0.5 μM P conditions (P_0.5_-Si), (ii) tea seedlings inoculated with *S*. *indica* under 0.5 μM P conditions (P_0.5_+Si), (iii) tea seedlings inoculated without *S*. *indica* under 50 μM P conditions (P_50_-Si), and (iv) tea seedlings inoculated with *S*. *indica* under 50 μM P conditions (P_50_+Si), respectively. Each treatment replicated eight times, in a total of 32 pots, in a completely randomized arrangement.

### Measurements of growth and root fungal colonization rate

Plant height, stem diameter, leaf number, shoot biomass, and root biomass were measured at plant harvest. The root fungal colonization was stained as per the protocol described by Yang L. et al. (2021), in which 1-cm root segments were incubated with 10% KOH solution at 90°C for 2 h, bleached with 10% hydrogen peroxide solution for 15 min, acidified with 0.2 mol/L HCl solution for 10 min, and stained with 0.05% trypan blue in lactophenol solution for 35 s. The fungal colonization of roots was observed under a microscope, and then the percentage of the number of fungus-colonized root segments versus the total number of observed root segments was set as the root fungal colonization rate.

### Measurements of leaf gas exchange, nitrogen balance index, and chlorophyll fluorescence parameters

In the morning of a sunny day before harvest, leaf gas exchange parameters, including net photosynthetic rate (Pn), intercellular CO_2_ concentration (Ci), stomatal conductance (Gs), and transpiration rate (Tr), were determined by a Li-6400 photosynthetic apparatus (LI-COR Inc., Lincoln, NE, USA) according to the user’s guide. Then, leaves were measured by a portable Plant Polyphenol-Chlorophyll Instrument (Dualex, Force-A company of France, Orsay, France), and chlorophyll index (Chl) and nitrogen balance index (Nbi) were obtained.

Chlorophyll fluorescence parameters including Fv/Fm_Lss (the maximum efficiency of PSII), QY_max (the maximum mass quantum yield), QY_Lss (the minimum quantum yield) and NPQ_Lss (the non-photochemical quenching) were determined in leaves by a high-throughput plant fluorescence phenotype monitoring platform (RAP-FLUO) of Gufeng Optoelectronic Co., Ltd., Wuhan, Hubei, China.

### Measurement of leaf P and endogenous hormones

A 0.3 g dry sample of leaves was digested with nitric acid/hydrogen peroxide (Wheal et al., 2011) and centrifuged at 300×*g*/min for 5 min. The supernantant was fixed and diluted to determine leaf P concentrations the inductively coupled plasma optical emission spectrometry (ICP-OES) (IRIS Advantage, Thermo, Waltham, MA, USA).

According to the protocol of Liu RC et al. (2021), indole acetic acid (IAA), indole butyric acid (IBA), dihydrozetin (DZ), isopentenyladenine (IP), and trans-zetin (TZ) were extracted from leaves and analyzed by the High Performance Liquid Chromatograph (LC-100, Shanghai Precision Instruments, Shanghai, China) (Chen et al., 2009) with a C18 column (250 mm × 4.6 mm × 5 μm), 0.8 mL/min flow rate through the extraction column, 35°C of the column temperature, 40 minutes of the detection time, and 254 nm of the detection wavelength.

### Analysis of relative expression of two *PT* genes in leaves

The 0.1 g leaf fresh samples were ground in liquid nitrogen, and total RNA was extracted using the TaKaRa MiniBEST Plant RNA Extraction Kit. The primeScript™ RT kit and gDNA eraser were used for reverse transcription of RNA. We selected two high-affinity *PT* genes, including *CsPT1* and *CsPT4* as per the results of Xin et al. (2017). Their primers were designed based on the online Genbank (http://www.ncbi.nlm.nih.gov/genbank/) and shown in the **Supplementary Table S1**. The expression of *CsPT1* and *CsPT4* in leaves was measured by quantitative real-time fluorescence PCR (qRT-PCR), and the relative gene expression was calculated using the method described by Livak and Schmittgen (2001), using the GADPH as an internal reference gene (Xin et al., 2017). The data were normalized to the expression of tea plants uninoculated with *S*. *indica* grown at the P_0.5_ level.

### Data analysis

Data were analyzed using the two-way analysis of variance (ANOVA) by SAS software (8.1v; SAS Institute Inc., Cary, NC, USA), and significant differences between treatments were compared with Duncan’s multiple range tests at *P* < 0.05.

## Results

### Changes in root fungal colonization rate

**Figure 1:**
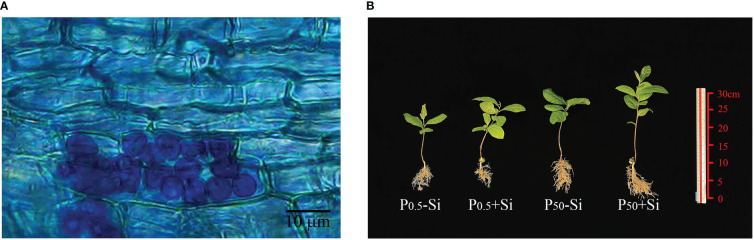
Root colonization of *Serendipita indica* **(A)** and plant growth responses **(B)** of tea seedlings inoculated with *S*. *indica* at P_0.5_ and P_50_ levels. The abbreviations are shown in **Table 1**.

No fungal colonization was found in the roots of tea seedlings uninoculated with *S*. *indica*, while the roots of tea seedlings inoculated with *S*. *indica* were colonized to varying degrees (**Figure 1A**), with fungal colonization rates ranging from 62.18% at P_0.5_ levels to 81.34% at P_50_ levels (**Table 1**). The root fungal colonization rate under P_50_ conditions was 0.31-fold significantly higher than under P_0.5_ conditions. There was a significant interaction in root fungal colonization rate between *S*. *indica* inoculations and P treatment.

**Table 1:** Effects of inoculation with *Serendipita indica* on root fungal colonization and plant growth parameters of tea seedlings at P_0.5_ and P_50_ levels.

Treatments	Root fungal colonization rate (%)	Plant height (cm)	Stem diameter (cm)	Number of blades	Shoot biomass (g/plant)	Root biomass (g/plant)
P_0.5_-Si	0 ± 0c	9.4 ± 1.27c	1.80 ± 0.50b	5.3 ± 1.5c	1.63 ± 0.15d	1.17 ± 0.16d
P_0.5_+Si	62.18 ± 6.57b	13.16 ± 2.33b	2.24 ± 0.40a	8.1 ± 1.9b	2.59 ± 0.24b	2.31 ± 0.13b
P_50_-Si	0 ± 0c	14.67 ± 1.44ab	2.50 ± 0.39a	8.9 ± 1. 6b	2.28 ± 0.22c	1.66 ± 0.17c
P_50_+Si	81.34 ± 4.56a	15.63 ± 2.98a	2.62 ± 0.37a	10.4 ± 1.9a	3.52 ± 0.23a	2.71 ± 0.24a
*Significance*						
*S*. *indica*	<0.0001	<0.0001	0.0020	<0.0001	<0.0001	<0.0001
P treatments	<0.0001	0.0021	0.0153	0.0005	<0.0001	<0.0001
Interaction	<0.0001	0.0570	0.1017	0.3398	0.0752	0.5133

Data (means ± SD, *n* = 5) followed by different letters in the column indicate significant (*P* < 0.05) differences. -Si, inoculation without *S. indica*; +Si, inoculation with *S. indica*; P_0.5_, 0.5 μM P; P_50_, 50 μM P.

### Changes in plant growth behavior

Plant growth behavior including plant height, stem diameter, leaf number, shoot biomass, and root biomass was higher at P_50_ levels than at P_0.5_ levels (**Figure 1B**; **Table 1**). In addition, compared with *S*. *indica* uninoculation, *S. indica* inoculation significantly increased leaf number, shoot biomass, and root biomass by 17.00%, 54.39%, and 63.25% at P_50_ levels, respectively, along with no significant change in plant height and stem diameter (**Table 1**). Nevertheless, *S. indica* inoculation significantly elevated plant height, stem diameter, leaf number, shoot biomass, and root biomass by 40.00%, 24.44%, 52.16%, 58.90%, and 97.44% at P_0.5_ levels, respectively, compared with non-*S*. *indica* inoculation. Significant interaction did not appear on these growth variables.

### Changes in leaf gas exchange

P_0.5_ treatment significantly inhibited leaf Pn, Gs, Ci, and Tr, regardless of *S*. *indica* inoculation or not, compared with P_50_ treatment (**Table 2**). However, *S*. *indica* inoculation significantly improved these gas exchange variables. At the P_0.5_ level, *S*. *indica* significantly increased leaf Pn and Tr by 25.13% and 78.26%, respectively; at the P_50_ level, *S*. *indica* significantly elevated leaf Pn, Gs, and Tr by 56.40%, 69.45% and 132.14%, respectively, compared with non-*S*. *indica* treatment. Significant interaction between *S*. *indica* inoculation and P treatments appeared on Pn, Gs, and Tr.

**Table 2:** Effects of inoculation with *Serendipita indica* on leaf gas exchange parameters of tea seedlings at P_0.5_ and P_50_ levels.

Treatments	Pn (g/m^2^/h)	Gs (μmol/m/s)	Ci (μmol/mol)	Tr (g/m^2^/h)	
P_0.5_-Si	1.95 ± 0.36d	10.86 ± 4.62c	240.92 ± 43.77a	0.23 ± 0.10d
P_0.5_+Si	2.44 ± 0.25c	11.16 ± 5.55c	242.17 ± 30.42a	0.41 ± 0.10c
P_50_-Si	2.89 ± 0.37b	19.90 ± 4.11b	204.08 ± 18.65b	0.56 ± 0.11b
P_50_+Si	4.52 ± 0.88a	33.72 ± 18.31a	180.33 ± 48.31b	1.30 ± 0.15a
*Significance*					
*S*. *indica*	<0.0001	<0.0001	<0.0001	<0.0001
P treatments	<0.0001	0.0191	0.2369	<0.0001
Interaction	0.0005	0.0245	0.1896	<0.0001

Data (means ± SD, n = 5) followed by different letters in the column indicate significant (*P* < 0.05) differences. Pn, net photosynthetic rate; Ci, intercellular CO_2_ concentration; Gs, stomatal conductance; Tr, transpiration rate. Other abbreviations are shown in **Table 1**.

### Changes in leaf Chl and Nbi

**Figure 2:**
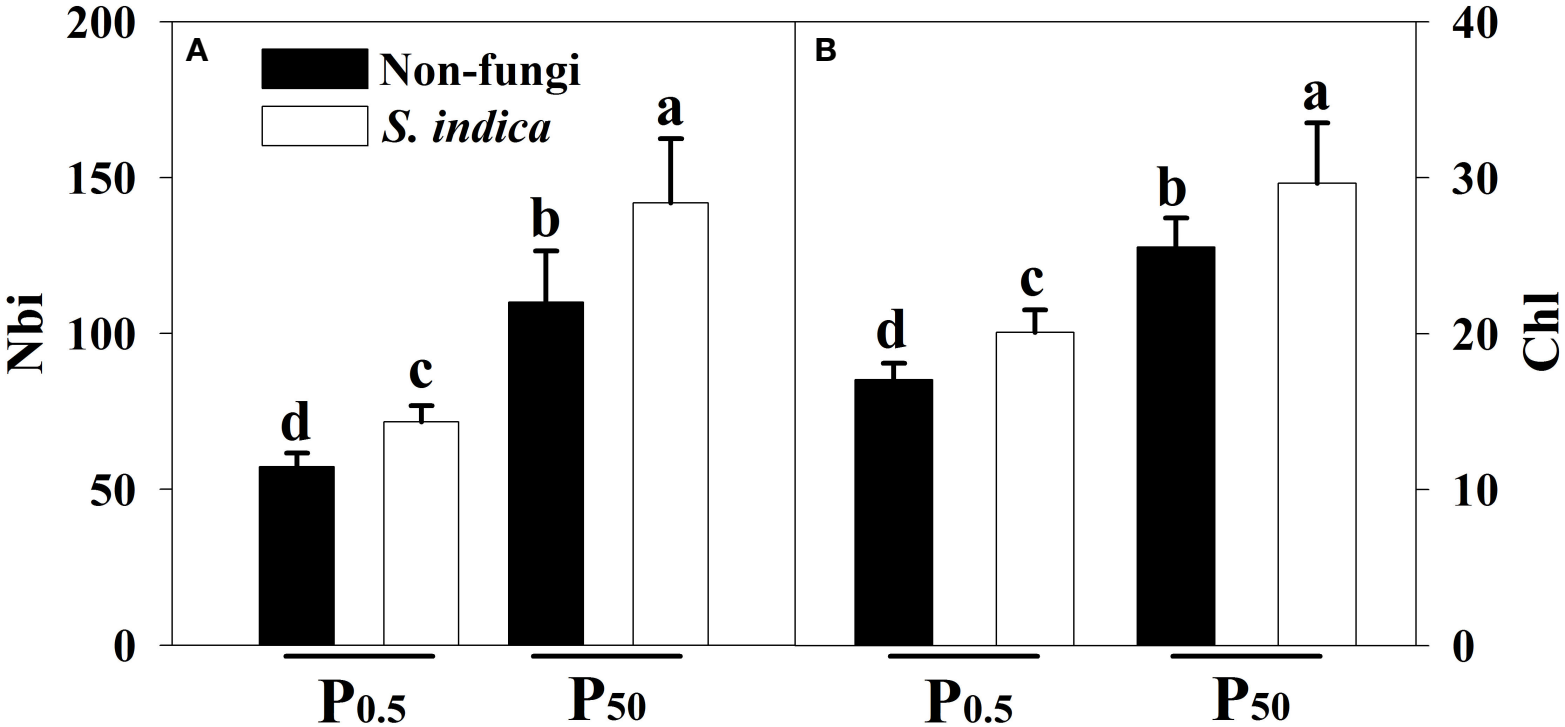
Effects of inoculation with *Serendipita indica* on nitrogen balance index (Nbi) **(A)** and leaf chlorophyll index (Chl) **(B)** of tea seedlings at P_0.5_ and P_50_ levels. Data (means ± SD, n = 5) are significantly (*P* < 0.05) different if followed by different letters above the bars. The abbreviations are shown in **Table 1**.

P_0.5_ treatment significantly inhibited leaf Chl and Nbi of inoculated and uninoculated tea plants, compared with P_50_ treatment: 47.59% and 98.03% lower in inoculated seedlings and 49.94% and 92.29% lower in uninoculated seedlings, respectively (**Figures 2A, B**). Compared with inoculation without *S*. *indica*, Nbi and Chl after *S*. *indica* inoculation significantly increased by 28.94% and 16.05% under P_50_ conditions and by 25.21% and 17.90% under P_0.5_ conditions, respectively. No significant interaction occurred in Chi and Nbi (**Table 3**).

**Table 3:** Factoral **s**ignificance in the interaction between *Serendipita indica* and P treatments.

Variables	*S*. *indica*	P treatments	Interaction	Variables	*S*. *indica*	P treatments	Interaction
Chl	<0.0001	0.0003	0.5458	TZ	<0.0001	<0.0001	0.0040
Nbi	<0.0001	<.0001	0.0817	DZ	0.1442	0.0857	0.0960
QY_Lss	0.0005	0.0036	0.1482	IAA	<0.0001	<0.0001	0.0777
QY_max	0.0145	0.2363	0.3303	IBA	0.1706	0.0003	0.0096
NPQ_Lss	0.0039	0.0223	0.3739	P	0.0001	0.0005	0.8926
Fv/Fm_Lss	0.0008	0.0277	0.0277	*CsPT1*	<0.0001	0.0001	0.1094
IP	<0.0001	0.0569	<0.0001	*CsPT14*	0.0002	0.0785	0.0951

**Figure 3:**
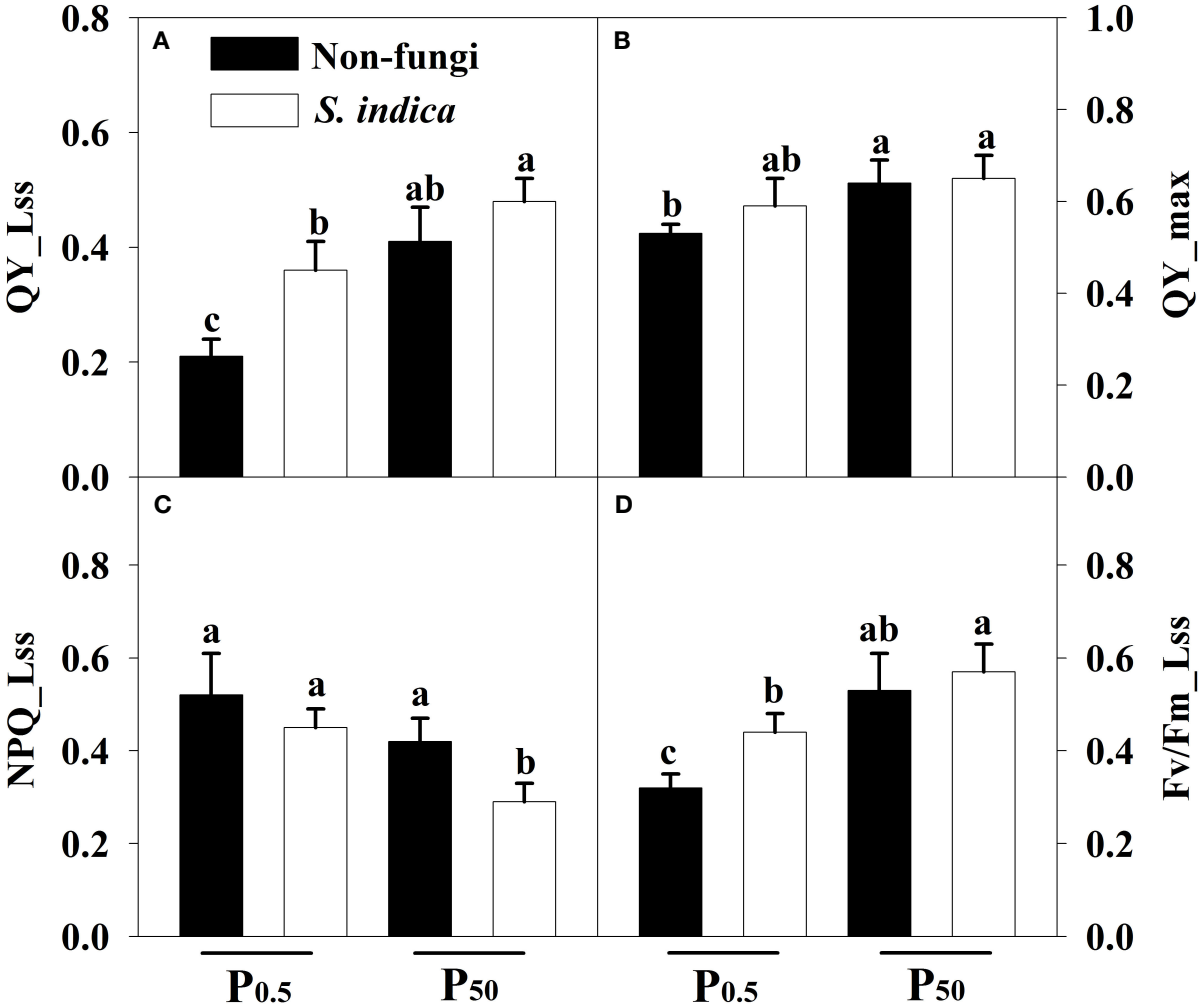
Effects of inoculation with *Serendipita indica* on leaf QY_Lss **(A)**, QY_max **(B)**, NPQ_Lss **(C)**, and Fv/Fm_Lss **(D)** of tea seedlings at P_0.5_ and P_50_ levels. Data (means ± SD, *n* = 5) are significantly (*P* < 0.05) different if followed by different letters above the bars. Fv/Fm_Lss, the maximum efficiency of PSII; QY_max, the maximum mass quantum yield; QY_Lss, the minimum quantum yield; NPQ_Lss, the non-photochemical quenching. Other abbreviations are shown in **Table 1**.

**Figure 4:**
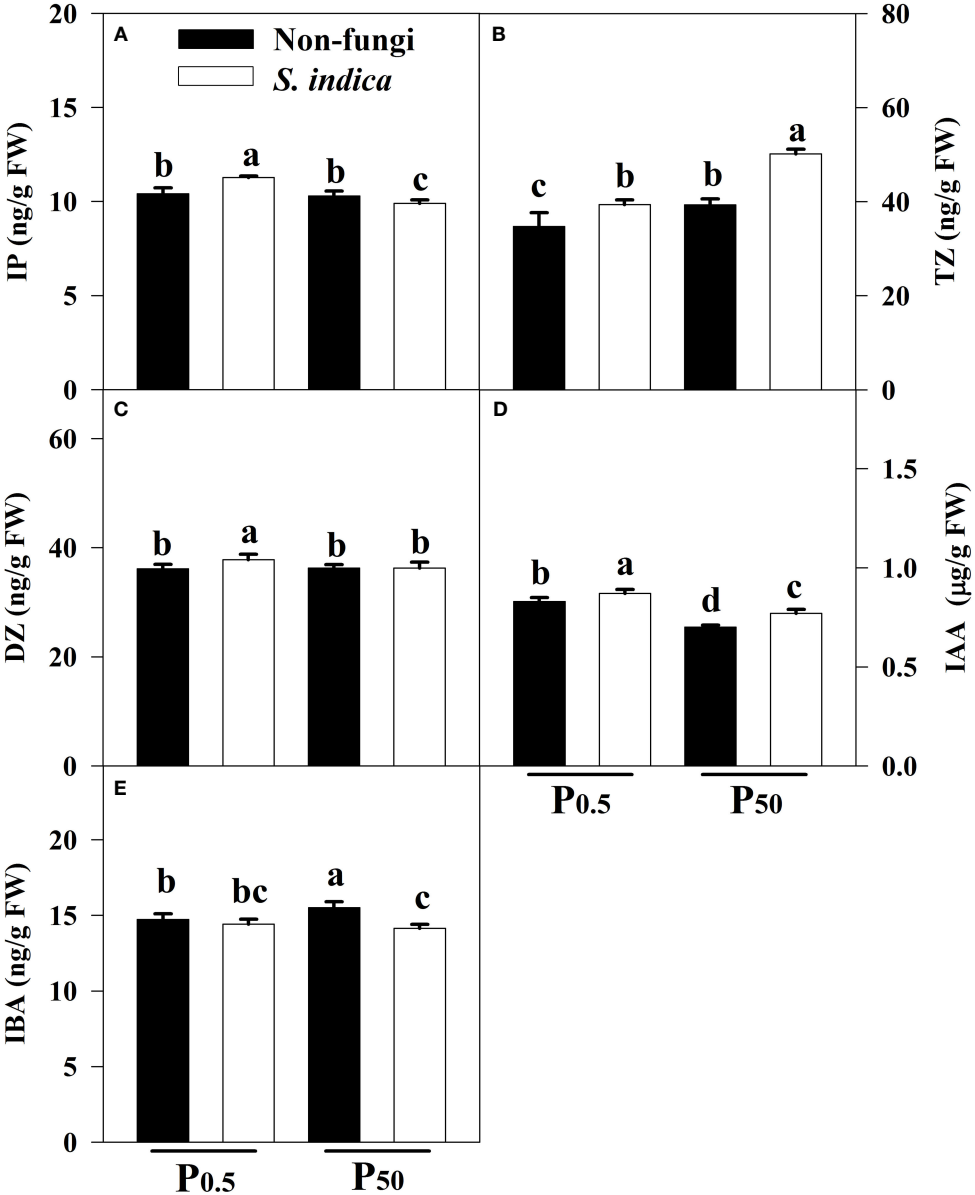
Effects of inoculation with *Serendipita indica* on leaf IP **(A)**, TZ **(B)**, DZ **(C)**, IAA **(D)**, and IBA **(E)** of tea seedlings at P_0.5_ and P_50_ levels. Data (means ± SD, *n* = 5) are significantly (*P* < 0.05) different if followed by different letters above the bars. IAA, indole acetic acid; IBA, indole butyric acid; DZ, dihydrozetin; IP, isopentenyladenine; TZ, trans-zetin. Other abbreviations are shown in **Table 1**.

**Figure 5:**
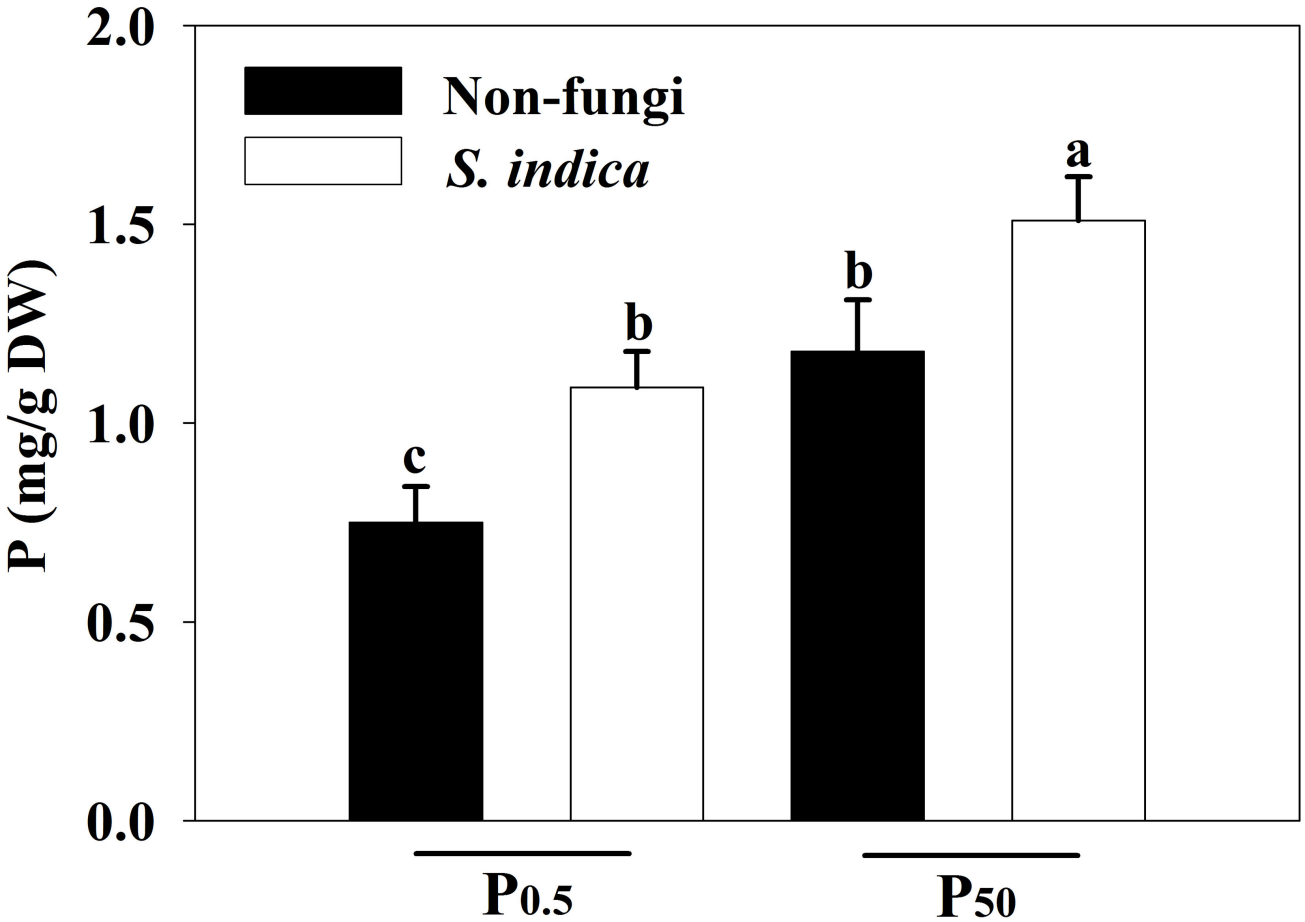
Effects of inoculation with *Serendipita indica* on leaf P concentrations of tea seedlings at P_0.5_ and P_50_ levels. Data (means ± SD, *n* = 5) are significantly (*P* < 0.05) different if followed by different letters above the bars. P, phosphorus. Other abbreviations are shown in **Table 1**.

**Figure 6:**
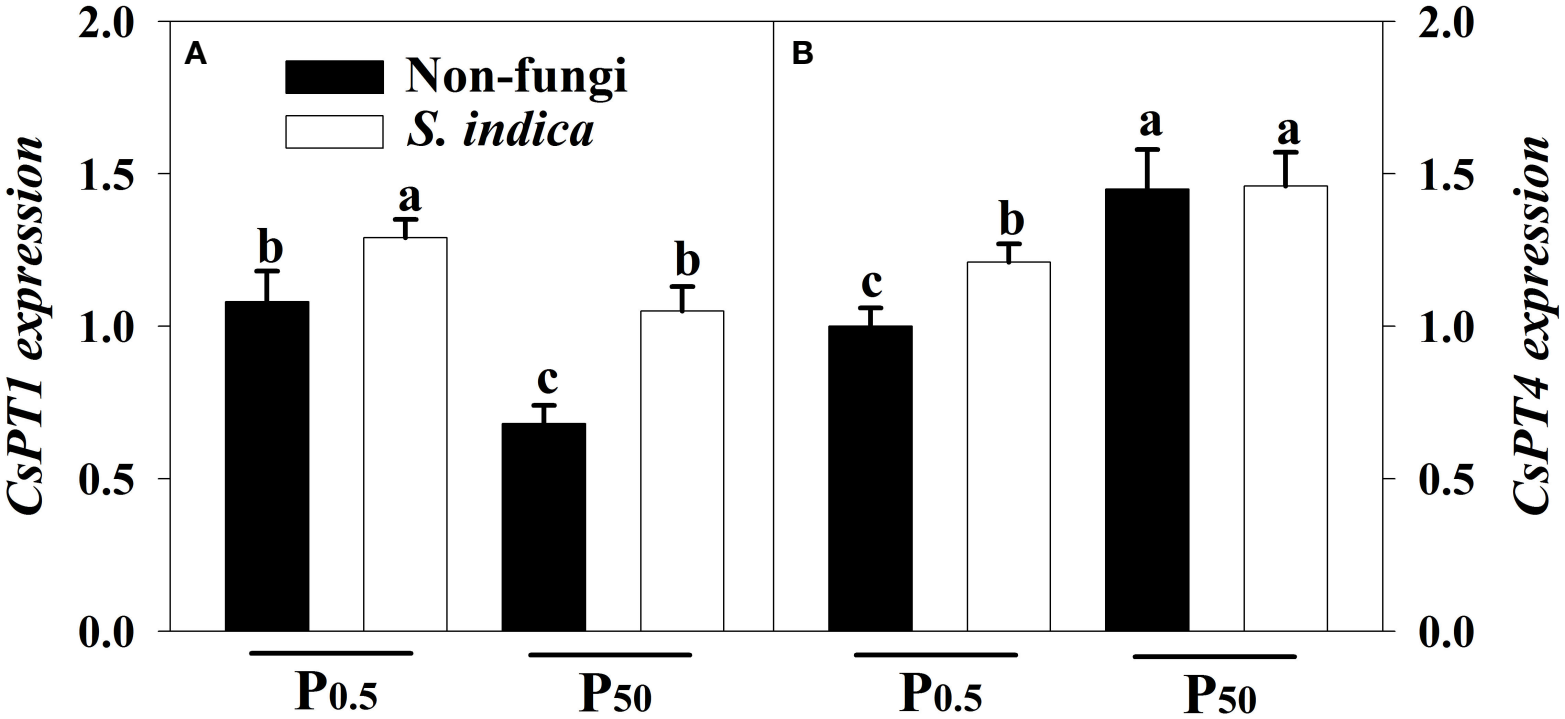
Effects of inoculation with *Serendipita indica* on leaf *CsPT1* **(A)** and *CsPT4* **(B)** expressions of tea seedlings at P_0.5_ and P_50_ levels. Data (means ± SD, *n* = 3) are significantly (*P* < 0.05) different if followed by different letters above the bars. The abbreviations are shown in **Table 1**.

The abbreviations are shown in **Figures 2**−**6**.

### Changes in leaf chlorophyll fluorescence parameters

P_0.5_ treatment significantly suppressed Fv/Fm_Lss, QY_max, and QY_Lss in uninoculated seedlings, compared to P_50_ treatment (**Figures 3A–D**). Similarly, inoculated plants showed lower Fv/Fm_Lss and QY_Lss while higher NPQ_Lss at the P_0.5_ level than at the P_50_ level. Compared with the uninoculated treatment, inoculation with *S*. *indica* showed a significant decrease in NPQ_Lss by 26.47% at P_50_, coupled with no significant changes in Fv/Fm_Lss, QY_max, and QY_Lss; Fv/Fm_Lss and QY_Lss increased significantly by 37.5% and 71.43% at P_0.5_ after inoculation with *S*. *indica*, but QY_ max and NPQ_Lss did not change significantly. A significant interaction occurred in Fv/Fm_Lss (**Table 3**).

### Changes in leaf endogenious hormones

Compared with the P_50_ treatment, the P_0.5_ treatment significantly increased IAA levels in leaves of uninoculated seedlings by 18.57% and decreased leaf IBA and TZ levels by 5.22% and 13.38%, respectively, along with no significant difference in leaf IP and DZ levels (**Figures 4A–E**). Similarly, compared with the P_50_ treatment, the P_0.5_ treatment significantly increased leaf IP, DZ and IAA levels of inoculated seedlings by 13.94%, 4.19% and 12.99%, respectively, and decreased leaf TZ levels by 27.44%, coupled with no significant change in leaf IBA levels. At the P_0.5_ level, inoculation of *S*. *indica* significantly increased leaf IP, DZ, TZ, and IAA levels by 8.36%, 4.59%, 13.46%, and 4.82%, respectively, accompanied by no significant change in leaf IBA levels, as compared with uninoculation of *S*. *indica*. At the P_50_ level, inoculation of *S*. *indica* significantly increased leaf TZ and IAA levels by 27.53% and 10.00%, respectively, but significantly decreased leaf IP and IBA levels by 4.04% and 9.61%, with no significant effect on leaf DZ levels, as compared with uninoculation of *S*. *indica*. In addition, there was a significant interaction at the IP, TZ and IBA levels between *S*. *indica* inoculation and P treatments (**Table 3**).

### Changes in leaf P concentrations

P_0.5_ treatment significantly reduced leaf P concentrations by 57.33% in uninoculated plants and 38.53% in inoculated plants, respectively, compared with P_50_ treatment (**Figure 5**). Additionally, compared with non-*S*. *indica* inoculation, *S*. *indica* inoculation significantly increased leaf P concentrations by 27.97% in P_50_ and by 45.33% in P_0.5_, respectively. Significant interaction did not appear on leaf P concentrations (**Table 3**).

### Changes in leaf *CsPTs* expression

The expression of *CsPT1* and *CsPT4* genes in leaves of tea seedlings was regulated by soil P levels and *S*. *indica* (**Figures 6A, B**). Compared with the P_50_ treatment, the P_0.5_ treatment significantly induced the up-regulated expression of *CsPT1*, but suppressed the expression of *CsPT4*, irrespectively of *S*. *indica* inoculation or not. On the other hand, inoculation of *S*. *indica* significantly increased the expression of leaf *CsPT1* and *CsPT4* under P_0.5_ conditions by 0.16- and 0.17-fold, respectively. However, inoculation of *S*. *indica* under P_50_ conditions only up-regulated leaf *CsPT1* expression by 0.31-fold, plus no significant change in *CsPT4* expression. Significant interaction did not appear on the two *CsPTs* expression (**Table 3**).

## Discussion

The symbiotic degree between plants and endophytic fungi is regulated by substrate P levels (Wu et al., 2015). In this study, the P_0.5_ treatment dramatically inhibited root colonization rate of *S*. *indica* in tea seedlings, compared to the P_50_ treatment, which may be due to the fact that under conditions of P deficiency, plants acquire P mainly through increased root-hair density (Shao et al., 2021), thus reducing the dependence on *S*. *indica*.

In the present study, we also found that leaf P concentrations were significantly higher under P_50_ conditions than under P_0.5_ conditions, and *S*. *indica* colonization promoted leaf P acquisition by tea seedlings under both P_0.5_ and P_50_ conditions, which is in agreement with the findings of Taghinasab et al. (2018) on wheat. *S*. *indica* improved plant growth behavior of tea seedlings to varying degrees, with a prominent positive effect appeared at the P_0.5_ level than at the P_50_ level. This suggests that the presence of endophytic fungi, such as *S*. *indica*, plays a crucial role in maintaining good plant growth under the condition of substrate P deficiency (Poveda et al., 2021). This is consistent with the results of previous studies in wheat (Taghinasab et al., 2018) and sunflower (Eliaspour et al., 2020).

P is an important macronutrient in plants that plays an important role in maintaining photosynthetic electron transfer efficiency as well as ribulose-1,5-bisphosphate carboxylase (Rubisco) activity (Nguyen et al., 2022). In the present study, we found that P_50_ promoted leaf Pn, Gs, and Tr in tea, compared with P_0.5_, showing that leaf gas exchange in tea seedlings is dependent on the level of substrate P. On the other hand, inoculation of *S*. *indica* also significantly increased leaf Pn and Tr at P_0.5_ and P_50_ levels. This is in agreement with the results of Sun YL et al. (2022) on alfalfa. Generally, P supply can accelerate leaf Pn and Tr (Pang et al., 2018). We also found that increased substrate P levels as well as *S*. *indica* inoculation significantly increased leaf Chi levels and Nbi, thus suggesting that the endophytic fungus stimulate photosynthetic activity by increasing Chl content as well as N levels to improve plant photosynthetic capacity (Liu et al., 2019). Hallasgo et al. (2020) also reported that inoculation of *S*. *indica* on 3-week-old tomato seedlings promoted N concentrations. These results demonstrated that *S*. *indica* improved nutrient uptake as well as Chl formation and gas exchange in tea seedlings (Mathur et al., 2018).

Chlorophyll fluorescence can reflect the absorption, utilization, transfer and dissipation processes of light energy by PSI and PSII (Cao YW et al., 2022). In this study, under *S*. *indica*-uninoculated conditions, compared with P_0.5_, Fv/Fm_Lss, QY_max, and QY_Lss increased significantly under P_50_ conditions, which is consistent with Yang YM et al. (2021) on soybean. It confirmed that chlorophyll fluorescence parameters are closely related to P levels. In addition, NPQ is an important indicator for plant adaptation to low P conditions (Yang YM et al., 2021). P_0.5_ significantly promoted NQP_Lss in *S*. *indica*-inoculated tea seedlings, compared with P_50_, further indicating that *S*. *indica*-inoculated plants have better ability to adapt to low P than uninoculated plants. Fv/Fm_Lss is an important indicator to determine whether plants are subject to photoinhibition (Chen et al., 2022). In this study, P_0.5_ treatment resulted in a photoinhibition in tea seedlings, whereas inoculation with *S*. *indica* significantly increased Fv/Fm_Lss at the P_0.5_ level, indicating that the photoinhibition of inoculated plants was somewhat alleviated and that photosynthetic organs could effectively convert absorbed light energy into biochemical energy, thus maintaining normal photosynthetic efficiency. Similar results were also reported by previous studies (Liu RC et al., 2021; Sun YL et al., 2022). Thus, inoculated tea seedlings with the endophytic fungus were able to slow down photoinhibition and promote light energy efficiency, thus maintaining efficient photochemical efficiency, especially at the P_0.5_ level. It reveals an important role of *S*. *indica* in tea seedlings growth under soil P deficit, but the exact mechanism is still unclear, and further deciphering how *S*. *indica* is involved in the host’s photochemical regulation of efficiency.

Plants regulate their growth and development through changing hormone levels to adapt to environments (Perez-Alonso et al., 2021), of which auxins and cytokinin are the main regulatory factors of plant growth and development (Bielach et al., 2017). In this study, two auxins, IAA and IBA, were measured and it was found that under uninoculated conditions, leaf IAA levels were significantly decreased under P_50_ conditions compared to P_0.5_, which is consistent with previous findings (Huang et al., 2022). While, IBA levels were significantly increased at P_50_ versus P_0.5_ levels. IBA is the storage form and source of IAA, which is more stable than IAA (Kunkel and Harper, 2018). Inoculated tea seedlings with *S*. *indica* recorded significantly higher leaf IAA levels and relatively lower IBA levels than uninoculated seedlings in P_0.5_ and P_50_, which is consistent with the results of Zhang et al. (2019) in trifoliate orange seedlings inoculated with *Funneliformis mosseae*. Such change may be due to the inoculation of *S*. *indica* to promote peroxidase activity, thus accelerating the conversion of IBA to IAA (Frick and Strader, 2018; Cheng et al., 2020). These results confirmed that the promotion of plant growth by symbiotic fungi under nutrient deficiency is mainly related to its regulation of IAA. Cytokinins usually act synergistically with auxins to promote plant growth and development (Kieber and Schaller, 2018).

In this study, P_0.5_ significantly decreased only leaf TZ levels of inoculated and uninoculated seedlings, compared to P_50_. Under P-deficient conditions, plants maintain their P uptake by increasing cis-zeatin (CZ) levels, which inhibits the synthesis of TZ, thus increasing the ratio of CZ/TZ to maintain its response to the cytokinin signaling pathway for facilitating plant P acquisition (Silva-Navas et al., 2019). In the present study, leaf IP, TZ, and DZ levels of P_0.5_-treated tea seedlingss were significantly increased after inoculation with *S*. *indica*, which is due to the combination of *S*. *indica* and low P promoting isopentenyl transferase (an important rate-limiting enzyme for cytokinin biosynthesis that catalyzes the breakdown of isopentenyl pyrophosphate and adenosine monophosphate to produce isopentenyl adenosine monophosphate as a precursor of cytokinins) activity (Liu et al., 2020), thus inducing cytokinin production in young tea seedlings.

In addition, *S*. *indica* can promote the acquisition of P in plants by inducing the expression of *PT* genes (Yang L et al., 2021; Cao MA et al., 2022). In tea, *CsPT1* and *CsPT4* belong to the Pht1 family and are mainly responsible for absorbing inorganic phosphate from soil (Xin et al., 2017). It was documented that an arbuscular mycorrhizal fungus, *Claroideoglomus etunicatum*, could induce the up-regulation of *CsPT1* gene and promote P absorption in tea plants grown in both P_0.5_ and P_50_ (Shao et al., 2021). In this study, *CsPT1* expression in young leaves was increased, while *CsPT4* expression was decreased under the condition of P_0.5_ versus P_50_. Xin et al. (2017) observed that low P induced the up-regulated expression of *CsPT4* in roots, along with higher expression of *CsPT4* in old leaves than roots. It is necessary to comprehensively analyze the response pattern of *CsPTs* to low P in old leaves, young leaves, stems, and roots. The present study also indicated *S*. *indica* colonization led to up-regulation of *CsPT1* expression under P_0.5_ and P_50_ conditions and *CsPT4* expression under P_0.5_ conditions. This suggests that *S*. *indica* up-regulates the expression levels of *CsPT1* and *CsPT4* at low P levels, further revealing the importance of endophytic fungi under nutrient deficit. At the appropriate P level, *S*. *indica* only increased the expression level of *CsPT1*, and had no effect on the expression of *CsPT4*. In fact, in *S*. *indica*, a high-affinity phosphate transporter *SiHPO80* could be up-regulated under the condition of appropriate P levels (Loha et al., 2018), which may compensate for the unresponsive expression of *CsPT4* at the appropriate P level. Moreover, the expression of *CsPT1* was up-regulated not only by *S*. *indica* but also by the arbuscular mycorrhizal fungus *C*. *etunicatum*, both at the P_0.5_ and P_50_ levels (Shao et al., 2021), which reveal that *CsPT1* is up-regulated by beneficial fungi, but whether its expression is specifically induced remains to be further verified. In addition, *S*. *indica* also releases acid phosphatase into the environment (Kushwaha and Kumar, 2022), thus dissolving insoluble P in the soil for enhanced P acquisition of the host. It is suggested that *S*. *indica* can improve host P acquisition through a variety of ways, such as up-regulation of host *PTs* expression and self-release of acid phosphatase and *SiHPO80* expression. However, it requires assessing how such multiple ways work at different P levels.

## Conclusion

The symbiosis between *S*. *indica* and tea seedlings was regulated by soil P levels, and an appropriate soil P level was favorable for symbiotic formation in roots. At the P_0.5_ level, inoculation of *S*. *indica* promoted P acquisition and thus plant growth in tea seedlings by regulating the expression of *CsPT1* and *CsPT4* genes as well as increased chlorophyll, gas exchange, and endogenous IAA, IP, and DZ levels. Such results provide a new pathway for future inoculation of tea seedlings with a culturable endophytic fungus *S*. *indica* in the field. However, the mechanism on how *S*. *indica* promotes P acquisition of tea plants at low P levels needs to be further investigated.

## Data availability statement

The original contributions presented in the study are included in the article/**Supplementary Material**. Further inquiries can be directed to the corresponding authors.

## Author contributions

Conceptualization, Q-SW and TY; data curation, Z-YR and A-QL; methodology, Z-YR; resources, Q-SW; supervision, Q-SW and TY; writing—original draft, Z-YR; writing—review and editing, AH, KK, EA, and Q-SW. All authors contributed to the article and approved the submitted version.
