# Supplementary material for: Serendipita indica promotes P acquisition and growth in tea seedlings under P deficit conditions by increasing cytokinins and indoleacetic acid and phosphate transporter gene expression
Source: Front Plant Sci. 2023 Mar 17;14:1146182. doi: 10.3389/fpls.2023.1146182 (PMC10064445; doi:10.3389/fpls.2023.1146182)
Supplement: Supplementary file 1 [file Table_1.docx]

**Supplementary Table S1** The specific primers of genes used the gene expression analysis.

| Gene name | Accession | Forward primer (5′→3′) | Reverse primer (5′→3′) |
| --- | --- | --- | --- |
| *GADPH* | XM_002263109 | TTGGCATCGTTGAGGGTCT | CAGTGGGAACACGGAAAGC |
| CsPT1 | KY886916 | GTTTACATTCTTGGTGCCTGA | TAAACCGGAACTGTCCTAATG |
| CsPT4 | KY132100 | ACACGCTGATATTGCTTGGAT | ACGAACCTCTGCTGCTCCTGG |
